# Cold type autoimmune hemolytic anemia- a rare manifestation of infectious mononucleosis; serum ferritin as an important biomarker

**DOI:** 10.1186/s12879-019-3722-z

**Published:** 2019-01-18

**Authors:** Chinthana Dematapitiya, Chiara Perera, Wajira Chinthaka, Solith Senanayaka, Deshani Tennakoon, Anfas Ameer, Dinesh Ranasinghe, Ushani Warriyapperuma, Suneth Weerarathna, Ravindra Satharasinghe

**Affiliations:** 1 Sri Jayawardanapura General Hospital, Thalapathpitiya, Nugegoda, Western province Sri Lanka; 2 Colombo South Teaching Hospital, Kalubowila, Dehiwala, Western province Sri Lanka

**Keywords:** Infectious mononucleosis (IMN), Hemolytic anemia, Ferritin

## Abstract

**Background:**

Infectious mononucleosis is one of the main manifestations of Epstein – Barr virus, which is characterized by fever, tonsillar-pharyngitis, lymphadenopathy and atypical lymphocytes. Although 60% of patients with IMN develop cold type antibodies, clinically significant hemolytic anemia with a high ferritin level is very rare and validity of serum ferritin as an important biomarker has not been used frequently.

**Case presentation:**

18-year-old girl presented with fever, malaise and sore throat with asymptomatic anemia, generalized lymphadenopathy, splenomegaly and mild hepatitis. Investigations revealed that she had cold type autoimmune hemolysis, significantly elevated serum ferritin, elevated serum lactate dehydrogenase level with serological evidence of recent Epstein Barr infection. She was managed conservatively and her hemoglobin and serum ferritin levels normalized without any intervention following two weeks of the acute infection.

**Conclusion:**

Cold type autoimmune hemolytic anemia is a rare manifestation of infectious mononucleosis and serum ferritin is used very rarely as an important biomarker. Management of cold type anemia is mainly supportive and elevated serum ferritin indicates severe viral disease.

## Background

Epstein – Barr virus is one of the most ubiquitous human viruses, infecting more than 95% the adult population worldwide. Infectious mononucleosis is the main clinical syndrome of Epstein – Barr virus infection; this clinical syndrome is characterized by fever, tonsillar-pharyngitis, lymphadenopathy and atypical lymphocytes.

In cold type autoimmune hemolytic anemia (AIHA) IgM antibodies are formed against the polysaccharide antigens of red blood cells, which cause agglutination in low temperatures leading to complement activation and hemolysis. Cold type AIHA is mainly caused by lymphoid malignancies and rarely it can be caused by infections mainly *Mycoplasma pneumoniae* and infectious mononucleosis (IMN). Diagnosis of cold type AIHA due to IMN is confirmed by demonstrating red cell aggregates in a peripheral blood smear, presence of high titers of cold antibodies, positive direct antiglobulin test (IgG negative / C3d positive) with serological evidence of recent IMN infection [1].

Although 60% of patients with IMN develop cold type antibodies, clinically significant hemolytic anemia with a high ferritin level is very rare [2]. There are only few published cases in medical literature; hence we describe a case of clinically significant cold type autoimmune hemolytic anemia associate with significantly high serum ferritin as a rare manifestation of IMN.

## Case presentation

Eighteen-year old girl presented to us with sore throat, malaise, fatigue and fever for 10 days. She was apparently well 10 days back where she initially developed a sore throat followed by fever with chills and a non-productive cough. She did not have any abdominal pain, acrocynosis or exertional dyspnea. She did not have any contact history of similar illness.

On examination she was well looking. She was pale but not icteric. Oral examination revealed inflamed tonsils. She had tender, discrete, mobile bilateral anterior and posterior cervical lymph nodes with bilateral inguinal lymphadenopathy. On admission she had fever (39.2 °C) with no evidence of dehydration. She did not have any evidence of peripheral gangrene or acrocynosis She was found to have tachycardia (110 beats/min) with normal blood pressure. Her cardiovascular and respiratory system examinations were unremarkable. Her abdominal examination revealed mild, non-tender splenomegaly with no hepatomegaly.

On admission laboratory testing was remarkable for macrocytic moderate anemia with a hemoglobin of 8.6 g/dl (normal 11-16 g/dl), mean corpuscular volume 96.3 fl (normal 80-96 fl), mean corpuscular hemoglobin 37.7 pg (normal 27-34 pg), red blood cell mass 2.28 × 10^6^/ul (normal 3.5–5.5 × 10^6^/ul), white blood cell count 8.27 × 10^3^/ul (normal 4–11 × 103), neutrophils 48.4% and lymphocytes 45.9%. Her blood pictures revealed macrocytes, spherocytes, few polychromatics and lymphocytosis with atypical lymphocytes. Her direct anti globulin test (DAT) was positive and DAT profile revealed positive for C3d and negative for IgG. Her monospot test was positive and Epstein Barr virus (IgM) antibody was positive as well (EBV- viral capsid antigen (VCA)- IgM using ELISA method was positive but IgG was negative). Her biochemical tests revealed serum ferritin > 1650 ng/ml (6.9–282.5 ng/ml), serum iron 12.6 μmol/l (normal 5-33 μmol/l), total iron binding capacity (TIBC) 50.1 (normal 52.0–101.0), transferrin saturation 25.15%, serum lactate dehydrogenase (LDH) 1673u/l (normal 200-400u/l), serum heptoglobulin < 9 mg/dl (normal 30–200 mg/dl), Aspartate transaminase (AST) 124.5u/l (normal 0-31u/l), Alanine transferase (ALT) 46.7u/l (normal 7-35u/l), total bilirubin 1.3 mg/dl(normal 0.3–1.20) with an increased indirect fraction, alkaline phosphatase 77.4u/l (normal 30- 120u/l), serum creatinine 41.1 μmol/l (normal 0-97 μmol/l), C reactive protein 24 mg/l (normal < 6.0 mg/l), erythrocyte sediment rate 60 mm/hour (normal 0-10 mm/hour). Her anti-nuclear antibodies and rheumatoid factor was negative. Her ultrasound scan abdomen showed mild splenomegaly (11.3 cm × 6.0 cm) and transthoracic echocardiogram was normal.

She was given analgesics and advised to bed rest and avoid cold exposure and started on folic acid 5 mg daily. Since she was clinically not symptomatic for anemia, blood transfusion was not done. After 3 weeks her hemoglobin rose up to 10 g/dl and serum ferritin level reduced to 925 ng/ml and after six weeks follow up there was no lymphadenopathy on examination, her hemoglobin rose up to 11.5 g/dl, serum ferritin level normalized to 250 ng/ml and the repeat (VCA) – IgM was negative but (VCA)-IgG became positive.

## Discussion and conclusions

Cold type autoimmune hemolytic anemia (AIHA) is a rare manifestation of infectious mononucleosis (IMN) and serum ferritin is used very rarely as an important biomarker. In this case our patient had cold type AIHA, significantly high levels of serum ferritin with hepatitis and splenomegaly, high levels of lactate dehydrogenase (LDH) with fever and generalized lymphadenopathy caused by infectious mononucleosis.

The exact pathogenesis of IMN causing cold type AIHA is poorly understood. But according to suggested mechanisms initially there is formation of IgM antibodies to EBV. Then due to the molecular mimicry, IgM antibodies cross react with RBC polysaccharide antigens and result in formation of antigen-antibody complexes. This will activate the complement (C1) complex. Then it will activate C4/C2 complex forming C3 convertase, which produces C3b, which will deposit on the RBC membrane. In cold temperatures, when IgM is bound to the antigen, C3b will activate C5, forming membrane attack complexes, which will eventually cause intravascular hemolysis. But upon warming, IgM antibody dissociates from the RBC membrane but C3b will remain attached to RBC surface. Those RBC will be eventually destroyed in the reticulo-endothelial system, mainly spleen and causes extravascular hemolysis [3]. In our patient hemolysis was confirmed by an increased total bilirubin with a high indirect fraction, increased LDH and decrease level of heptoglobulin. Since direct antiglobulin test (DAT) became positive with DAT profile revealing positive C3b with a negative IgG, we confirmed she had cold type – autoimmune hemolytic anemia. Interestingly our patient did not have clinical acrocyanosis, so may be the extravascular hemolysis was predominant in her causing the moderate anemia.

In this case she had significantly high levels of serum ferritin with mild hepatitis and mild splenomegaly. Causes for her high serum ferritin may be multifactorial. It can be due to acute phase response following Epstein Barr infection. In our patient there was mild elevation of C reactive protein (CRP) but very high levels of serum ferritin. It has been shown that although classical inflammatory parameters such as CRP have proven its usefulness in bacterial or fungal infections, when it comes to viral infection CRP often has very low sensitivity and is non-discriminatory. It has also shown that during an acute Epstein Barr virus (EBV) infection there will be a rise in serum ferritin as an acute phase reactant and the ferritin level will rise according to the disease severity along with interleukin 8, whereas in most acute EBV infections CRP will be non-specific and will be below the detected level. This shows, although CRP and ferritin both are acute phase reactants formation of them involve two separate inflammatory pathways [4]. High serum ferritin also can be due to a chronic hemolytic anemia. Barbec V et.al has done measurements of serum ferritin levels of 121 patients who have been diagnosed to have various chronic hemolytic disorders and it has shown that there is a moderate rise in serum ferritin levels in autoimmune hemolytic anemia and the range of serum ferritin value is associated with the degree of hemolysis [5]. Mainly the ferritin rise due do hemolysis occurs in chronic hemolysis. But in our patient, it was mainly an EBV induced acute hemolysis, which occurred within 2 weeks. On top of this, research states that with an EBV infection itself, without hemolysis there is a rise in ferritin as an acute phase reactant with an average of 431 ng/ml [4]. Our patient’s serial values of serum ferritin gradually came to normal within 6 weeks with settling of the infection. Here what we emphasize is, in our patient, rise of serum ferritin was probably due to both hemolysis and as an acute phase response to the infection and nevertheless with or without hemolysis it’s important to do a serum ferritin level as a biomarker if someone clinically suspects an EBV infection. Serum ferritin can also be high in EBV associated hemophagocytic lymphohistiocytosis (HLA), but in our patient HLA was unlikely to occur because she did not fulfill the criteria for the diagnosis of HLA.

It has been found that 90% patients with acute EBV infection have asymptomatic deranged liver function tests and 50–75% have splenomegaly as in our index case. When deranged liver functions occur with high serum ferritin, acute EBV infection is also an important cause to exclude with more common causes such as hemochromatosis [6]. In this patient there are two main possible causes for asymptomatic deranged liver functions. Epstein Barr infection itself can cause mild hepatic damage. Exact mechanism is not very well understood but likely involves the host immune response to EBV antigens as suggested by Liver biopsy demonstrating a sinusoidal infiltrate of mononuclear cells, mixed portal tract inflammatory infiltrate and mild hepatocyte ballooning and vacuolization [7]. Secondly high ferritin levels can also contribute to mild hepatic damage as in our patient.

In this patient, she had very high levels of lactate dehydrogenase (LDH). Cause for this can be multifactorial. It can be due to the hemolysis, hepatitis or proliferation of lymphoid cells. LDH is an enzyme, which catalyzes the conversion of lactate into pyruvic acid. It has five iso-enzymes. LDH 1 and 2 are mainly located in red blood cells; LDH 3 is mainly located in lymphocytes and LDH 5 mainly in the liver. It has been shown that serial measurements of LDH are useful when evaluating the response to treatment in hemolysis because LDH level decreases with reduction of the hemolysis rate [8]. Mygind N et.al has done serial measurements of aspartate transaminase (AST) and LDH levels in 36 patients with sero-positive IMN and was found to have 70% of them having high AST and all of them having high LDH 1,2 or 3 iso-enzymes, but only 35% had elevated LDH 5 iso-enzyme. Furthermore after 10 weeks of acute infection AST levels became normal, but even after 16 weeks 50% of patients had elevated LDH [9]. So elevated LDH levels in our patient should be mainly due to proliferation of lymphoid cells and hemolysis, and it demonstrates the usefulness of monitoring serial LDH values in her because after recovery of the acute hemolysis episode, if she persists having high LDH levels then this could be due to ongoing lymphoid proliferation and she might possess a high risk of a lymphoid neoplasms later in her life.

Furthermore, although blood transfusion is a lifesaving procedure, sometimes it can result in life threatening complications. So, blood transfusion should be done only if it’s definitely indicated. Specially in a female who is in her child bearing age, as in our patient, transfusion can give rise to unwanted pregnancy related side effects such as hemolytic disease of new born (HDN). It has been found that majority of cases of HDN is due to non-Rh-D antibodies which has been sensitized by previous blood transfusions rather than sensitization due to previous pregnancies [10]. So, this shows the importance of conservative management of even a moderate anemia without blood transfusion in situations such as primary EBV infection.

We believe though, a rare manifestation, cold type autoimmune hemolytic anemia should be considered in infectious mononucleosis patients who are presenting with anemia. This case report highlights the importance of conservative management of anemia secondary to EBV infection specially in a child bearing age female, and also the value of serum ferritin as an initial biomarker since usual biomarkers such as C reactive protein levels remains within normal range during viral infections.

## Abbreviations

AIHA: Auto Immune Hemolytic Anemia
ALT: Alanine Transaminase
AST: Aspartate Transaminase
CRP: C Reactive Protein
EBV: Epstein Barr Virus
HDN: hemolytic Disease of Newborn
HLA: Haemophagocytic LymphoHistiocytosis
IMN: Infectious Mononucleosis
LDH: Lactate Dehydrogenase
RBC: Red Blood Cell
VCA: Viral Capsid Antigen
